# Clinicopathologic Features and Outcome of Adenocarcinoma of the Anal Canal: A Population-Based Study

**DOI:** 10.1155/2020/5139236

**Published:** 2020-05-13

**Authors:** Shekhar Gogna, Roberto Bergamaschi, Agon Kajmolli, Mahir Gachabayov, Aram Rojas, David Samson, Rifat Latifi, Xiang Da Dong

**Affiliations:** Division of Surgical Oncology, Westchester Medical Center, New York Medical College, 100 Woods Road, Taylor Pavilion, Office Suite #353, Valhalla, NY 10595, USA

## Abstract

**Background:**

Anal canal adenocarcinoma (AA) is an uncommon tumor of the gastrointestinal tract. We seek to provide a detailed description of the incidence, demographics, and outcome of this rare tumor in the United States.

**Methods:**

The data on anal canal adenocarcinoma from SEER Program, between 1973–2015, were extracted. We analyzed the incidence rates by demographics and tumor characteristics, followed by analysis of its impact on survival.

**Results:**

The incidence of AA increased initially by 4.03% yearly from 1973 to 1985 but had a modest decline of 0.32% annually thereafter. The mean age for diagnosis of AA was 68.12 ± 14.02 years. Males outnumbered females by 54.8 to 45.2%. Tumors were mostly localized on presentation (44.4%) and moderately differentiated (41.1%). Age generally correlated with poor overall cancer survival. However, young patients (age <40 years) also showed poor long-term survival. Patients with localized disease and well-differentiated tumors showed better survival outcomes. Surgical intervention improved survival significantly as compared to patients who did not (116.7 months vs 42.7 months, *p* < 0.01).

**Conclusions:**

Anal canal adenocarcinoma demonstrated a poor bimodal cancer-free survival in both younger and older patient groups. Surgery significantly improves odds of survival and should be offered to patients amenable to intervention.

## 1. Introduction

Adenocarcinoma of the anal canal is a rare neoplasm. Worldwide, the incidence is only a few thousand cases per year. Histologically, it represents approximately 16.5% of all types of anal canal cancers, which is dominated by squamous cell carcinoma [1]. The anal canal extends from the anal margin to the anorectal ring/flexure representing the terminal part of the gastrointestinal tract. Anatomically, based on the lining epithelium, the anal canal can be divided into the colorectal zone defined by the colorectal type of glandular mucosa proximally, the anal transition zone defined by the variable appearance of the lining epithelium in the middle, and the distal portion lined by squamous epithelium [1, 2].

Several proposals have been made as to the pathologic mechanisms leading to the anal canal adenocarcinomas (AA). These include anal glandular carcinomas originating from the anal glands, colloid carcinomas associated with Paget's disease of the anus, and adenocarcinomas arising from chronic fistula and inflammatory epithelium in the anus, as well as adenocarcinomas that arose from the distal rectum with extension into the anal canal [3, 4]. Previous observations that these malignancies were associated with chronic intestinal diseases such as preexisting fistulas or Crohn's disease prompted the hypothesis for the pathologic development of adenocarcinoma of the anal canal [3].

More recently, mutational analysis was able to differentiate anal canal adenocarcinoma into region-specific subtypes. The differences in HPV (16 and 18) infection status and expression of the immune checkpoint and mutational profile of several targetable genes separate this neoplasm into the 2 distinct entities: anal glandular/transitional subtype and colorectal subtype. From a treatment standpoint, anal glandular/transitional type cancers respond poorly to standard treatments. Mutational analysis showed that it harbored less frequent mutations in downstream factors of the EGFR signaling pathway, but a significantly higher expression of immune checkpoint inhibitors PD1/PD-L1 compared to its colorectal subtype counterpart. These tumors are Krt7 positive as opposed to Krt20 and CDX2 positive commonly seen with the colorectal subtype. The other colorectal subtype appears to be closely related to the tumors arising from the colorectal mucosa [5].

Anal canal adenocarcinomas are frequently thought to be more aggressive than squamous cell carcinomas [6, 7]. Traditional management of adenocarcinomas of the anal canal has relied on multimodal treatment to avoid local or distant failures. Currently, combined multimodality with radical surgical resection appears to portend more favorable prognosis [8]. Five-year overall survival is thought to exceed 60% following curative surgery in combination with chemoradiation [8, 9].

Because of the location of the tumor, the pattern of recurrence also differs from traditional rectal cancer [10]. Patients tend to have a higher incidence of lymph node metastasis in the groins necessitating concurrent management of the inguinal canal. With the recent advent of personalized genomic medicine, we seek to evaluate the epidemiology and overall prognosis of patients afflicted with this disease based on the large SEER database and hopes to provide insight on the patterns of treatment and failures.

## 2. Materials and Methods

This is a retrospective cohort study from the SEER database using 18 registries to identify all patients with anal canal adenocarcinoma from 1973–2015, using SEER site specific primary code, based on the International Classification of Diseases for Oncology, third edition (ICD-O-3). Anal canal cancers were classified by site: anal, not otherwise specified (NOS), (C21.0), anal canal (C21.1), and cloacogenic zone (C21.2). Patients with adenocarcinoma of anal canal overlapping with the rectum (C21.8) were excluded, as there is significant heterogeneity and difficulty to distinguish from patients with low lying rectal cancers. The histology codes used for adenocarcinoma were extracted from ICD-O-3 and are listed in Supplementary Table S1. The histological subtypes of various types of adenocarcinoma are listed in Supplementary Table S2.

### 2.1. Statistical Analysis

We used the frequency and survival session from SEER∗Stat version 8.3.5 to collect incidence, trend analysis, and 1 through 5-year survivals based on age, gender, location, histology, and malignant behavior. Patients with missing data were excluded from the analysis. Statistical analysis was conducted using SPSS for IBM Corp. (IBM SPSS Statistics for Windows, Released 2017, Version 25.0. Armonk, NY: IBM Corp). The overall survival (OS) rates were calculated using the actuarial (Kaplan–Meier) method. Differences in survival based on age, gender, histology, grade, and surgery were computed using a log rank test (Mantzel-Cox). Multivariate analysis was conducted using Cox regression analysis to identify the independent effect of cancer type on survival controlling for age, year of diagnosis, gender, race, tumor stage, and grade. All *p* values were 2-sided and the *p* value of <0.05 was considered significant.

According to the hospital policy, the institutional review board approved and exempted the study. This retrospective observational study is reported according to STROBE guidelines [11].

## 3. Results

There were 2090 patients with anal canal adenocarcinoma (AA). The median age for AA was 68.12 ± 14.02 years (range was 20–100 years). The disease showed male (*n* = 1145, 54.8%) preponderance as compared to females (*n* = 945, 45.2%). The disease, without normalizing to population demographics, was more commonly reported in whites (*n* = 1629, 77.9%), followed by blacks (*n* = 291, 13.9%) and all others (*n* = 170, 8.1%). Tumor grade ranged from Grade I well differentiated (*n* = 263, 12.6%), grade II moderately differentiated (*n* = 858, 41.1%), and grade III poorly differentiated (*n* = 340, 16.3%) to grade IV undifferentiated (*n* = 31, 1.5%) and cell type not determined (*n* = 598, 28.6%) (Supplementary Table S2). Based on the SEER database, the extent of disease was in situ (*n* = 141, 6.7%), localized (*n* = 927, 44.4%), regional (*n* = 539, 25.8%), and distant (*n* = 281, 13.5%), as well as incomplete information (*n* = 201, 9.6%).

### 3.1. Incidence Rates

**Figure 1:**
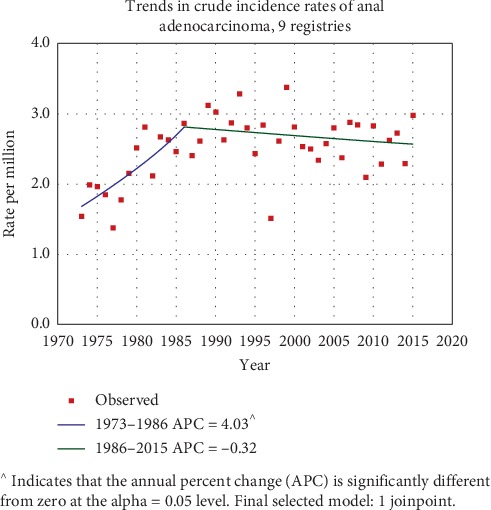
Changing incidence from 1973–2015.

The estimated current prevalence rate of adenocarcinoma of anal canal is 0.0011% as calculated from SEER∗Stat. The annual percentage change (APC) is used to measure trends or the change in rates over time. The Joinpoint software 6.0.0 was used to calculate the annual percentage change (APC) in incidence rates from 1973 to 2015. The incidence of AA increased annually by 4.03% from 1973 to 1985 and it showed a downward trend annually by 0.32% from 1986 to 2015. This rise and then decrease in annual incidence rate are shown in Figure 1. The reasons for this significant change in epidemiology remain to be investigated.

### 3.2. Survival

**Figure 2:**
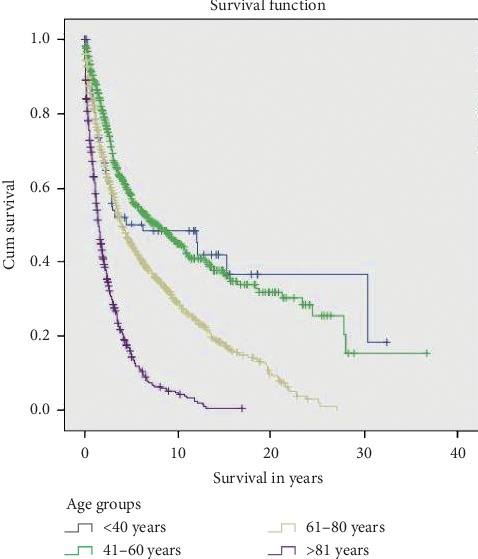
Kaplan–Meier plots showing overall survival among patients with anal canal adenocarcinoma (AA) based on age groups.

**Figure 3:**
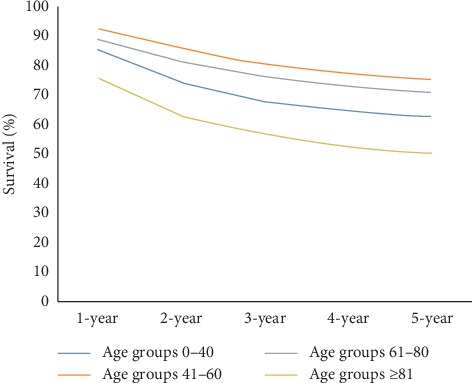
Trend analysis of cancer-free survival rates according to various age groups.

We stratified the patients into four age groups (<40 years, 41–60 years, 61–80 years, and ≥81 years). Elderly patients >81 years had the worst survival (mean survival of 30.14 ± 1.84 months) (HR, 3.79; 95% CI, 2.65–5.41) (*p* < 0.01)], as shown by the Kaplan–Meier Plot (Figure 2). Interestingly, patients who were younger than 40 years and elderly patients aged >81 years both showed initial poor cancer-specific survival rates. Patients in group II (41–60 years) had the best 1 through 5-year cancer-specific survival rate followed by patients in group III (61–80 years) (Figure 3). Eventually, group 1 patients (<40 years) showed improved long-term disease-free survival after 5 years. The calculated overall 1-, 2-, 3-, 4-, and 5-year survival rates are 76.1%, 63.4%, 52.6%, 47.9%, and 39.6%, respectively. Amongst patients with AA, males outnumbered females (54.8 vs 45.2%). The survival in males as compared to females was 98.8 vs 88.6 months, respectively, which was not statistically significant (*p*=0.13). Anal canal adenocarcinoma was also more common in whites (77.9%), although not normalized to the population. However, race was also not a statistically significant factor (*p*=0.06) influencing survival.

The primary site for anal canal cancer is divided into three zones according to ICD-O-3 morphology codes: anal, not otherwise specified (NOS), anal canal, and cloacogenic zone. We excluded cancers of anal canal overlapping with the rectum. The mean survival was highest in cancer of the anal canal with mean survival being 98.38 ± 5.56 months. However, location of cancer did not have statistical significance on long-term survival (*p*=0.405).

Anal canal adenocarcinoma had 12 various subtypes (not all listed); adenocarcinoma NOS (the general variety) was the most common subtype (Supplementary Table S2). The survival was best when AA originated in the polyps (172.90 ± 15.25 months), which was statistically significant (*p* < 0.01). This emphasizes the importance of carefully screening for the polyps in the anal canal.

**Figure 4:**
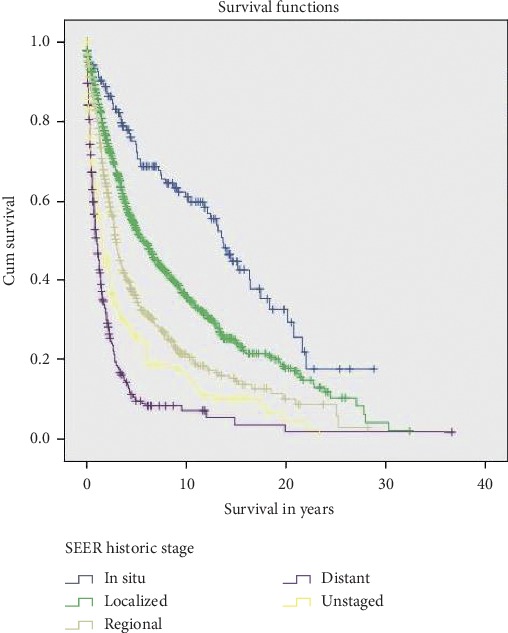
Kaplan–Meier plots showing overall survival among patients with anal canal adenocarcinoma (AA) based on pathologic stage.

As expected, the survival is best in patients who present with localized disease (mean survival in months 166.88 ± 12.62) as compared to patients presenting with distant metastasis (33.81 ± 6.024 months). Patients with metastatic disease have 6-fold mortality rate (HR, 6.02; CI, 4.55–7.99; *p* < 0.0001). The survival curve in Figure 4 shows that as with increasing stage of the disease, overall survival decreased. The survival characteristics are summarized in Table 1.

Table 2 shows the results of a multivariate Cox regression analysis among patients with AA to study the effect of various patient-related factors on survival. Increasing age (HR, 3.79; 95% CI, 2.65–5.41; *p* < 0.01), advanced stage of the disease (regional disease HR, 1.27; 95% CI, 1.08–1.49; *p* < 0.01) (distant disease HR, 2.8; 95% CI, 2.35–3.36; *p* < 0.01), and no surgical intervention (*p* < 0.01) significantly influenced the survival among patients with AA. There was a strong trend of improved survival among patients who received surgery.

## 4. Discussion

Anal canal adenocarcinoma is an uncommon diagnosis that portends poor overall survival [12]. The majority of anal canal cancers are squamous cell type carcinomas which are currently treated with chemoradiation [13]. Therefore, the management of AA is not standardized and fragmented in practice until recently, as proposed by NCCN guidelines [1]. Because of the rarity of this disease, most reports in the literature consisted of case series or case reports that are described in a retrospective fashion. Limited long-term follow-up data are available with regards to this unusual diagnosis. Because of the unfavorable outcome seen with this disease, there has been studies in the past suggesting that chemoradiation should be the only treatment option [14, 15]. Initial proposal by Papagikos et al. modelled the treatment similar to management of squamous cell carcinoma of the anal canal, albeit with poor outcomes with 5-year survival at 19% [14]. Subsequent studies from the MD Anderson and Memorial Sloan Kettering Cancer Center showed that combined modality including surgery can improve 5-year survival [9, 16]. These are some of the data that led to NCCN proposing a combined approach for management of this rare cancer in 2004.

There remains a lot of controversy in terms of management of this disease over the last decade. Belkacemi et al. analyzed the Rare Cancer Network and showed a respectable 5-year overall survival rate of 58% for patients treated with chemoradiation alone [15]. In contrast, a previous analysis of the SEER database by Franklin et al. concluded that those undergoing surgery showed improved survival compared with no surgical intervention [17]. However, some case series have shown that aggressive surgical resection along with chemoradiation may offer chances of long-term improved survival [8]. Li et al. analyzed the NCDB database and evaluated patients who have nonmetastatic and potentially curable disease and proposed that patients undergo surgery following chemoradiation as definitive treatment, with the most favorable 5-year survival of patients with AA published to date [8]. Their results are similar to the earlier SEER database analysis made by Franklin et al. [8, 17], As reflected in treatment of patients with gastrointestinal adenocarcinoma, AA patients with complete surgical resection appear to have the best long-term survival interval [18].

This review is the largest review to date with regards to AA. Based on the review of the SEER database with long-term follow-up, several takeaway messages can be formulated. First, this disease has a bimodal distribution with younger and older patients having poor prognosis initially. Second, surgical intervention can improve outcome, with potential for long-term survival. Because this disease is an adenocarcinoma, surgical intervention should be the cornerstone of treatment with concurrent chemoradiation when appropriate. However, due to its location, the metastatic pattern of this tumor follows that of anal canal squamous carcinoma. Therefore, nodal metastasis to the groin nodes can occur early and should be part of the initial treatment planning [19].

Our study did have few limitations including being retrospective in nature. There is also a degree of heterogeneity in the reported dataset as the patients with adenocarcinoma of the anal canal is not a well-recognized clinical entity. Divisions into the zones of involvement, histological subtypes, and appropriate staging of anal canal adenocarcinomas are all areas of controversy which lacks definitive clarity. Moreover, the details about chemotherapy regimen in the reported series were not available. Nonetheless, given that the pathology is quite rare, this represents a large data series that will hopefully shed light on an otherwise unrecognized disease entity. Any definitive treatment plan should be formulated or recommended based on multidisciplinary treatment planning following disease presentation.

## 5. Conclusion

On the basis of our analysis of anal canal adenocarcinoma from the SEER database, it appears that the incidence of AA is decreasing. The prognosis of the disease remains dismal in the elderly. The bimodal distribution of poor short-term cancer-free survival in younger and older patients is also evident. Surgical resection improves the chance of cure over chemoradiation alone. We do feel the need for multicenter studies on AA given the lack of adequate research in this field.

## Figures and Tables

**Table 1:** Survival characteristics of patients with anal canal adenocarcinoma.

Variable	Survival (months)	SD	95% CI	*p* value
Age				
<40 years	167.55	24.13	120.1–214.9	<0.01
41–60 years	162.85	11.00	141.2–184.4	
61–80 years	86.75	3.33	80.2–93.2	
≥81 years	30.14	1.84	26.5–33.7	

Gender				
Male	88.60	4.71	79.3–97.8	0.13
Female	98.84	4.62	89.7–107.9	

Race				
Whites	94.70	4.08	86.6–102.7	0.06
African Americans	78.66	6.99	64.9–92.3	
Asians/Pacific	131.03	29.75	72.7–189.3	
Others	104.08	10.38	83.7–124.4	

Stage				
In situ (0)	166.88	12.62	142.1–191.6	<0.01
Localized (1)	114.29	4.92	104.6–123.9	
Regional (2)	77.26	5.26	66.9–87.5	
Distant (3)	33.18	6.02	22.0–45.6	
Information not sufficient (9)	51.63	5.80	40.2–63.0	

Surgery				
Yes	116.74	4.58	107.7–125.7	<0.01
No	42.70	2.91	36.9–48.4	

**Table 2:** Cox hazard regression of predictors of survival.

Variable	HR	95% of HR	*p* value
Age			
0–40 years (reference)	1		
41–60 years	0.97	0.67–1.38	0.866
61–80 years	1.62	1.15–2.30	0.006
>81 years	3.79	2.65–5.41	<0.01

Race			
American Indians/Alaska Natives (reference)	1		
Whites	1.42	0.80–2.56	0.21
African Americans	1.62	0.90–2.90	0.29
Asians/Pacific Islanders	1.19	0.60–2.198	0.30

Gender			
Female (reference)	1		
Male	1.08	0.97–1.20	0.13

Stage			
In situ (reference)	1		
Localized only	0.87	0.75–1.00	0.06
Regional spread by direct extension only	1.27	1.08–1.49	0.03
Distant site(s)/node(s) involved	2.8	2.35–3.36	<0.01
Unknown	2.1	1.76–2.76	<0.01

Grade			
Well differentiated (reference)	1		
Moderately differentiated;	1.37	1.15–1.64	<0.01
Poorly differentiated; differentiated	2.15	1.76–2.63	<0.01
Undifferentiated; anaplastic	1.81	1.37–2.88	0.01
Cell type not determined	1.11	0.92–1.34	0.24

Surgery			
Yes (reference)	1		
No	0.38	0.34–0.42	<0.01

## Data Availability

The data supporting this SEER database analysis are available from the SEER database. All supplementary data are also included in the submitted manuscript. Alternatively, the data used to support the findings of this study are available from the corresponding author upon request.

## Supplementary Materials

Supplementary Materials Supplementary Table S1: histology coding classification. Supplementary Table S2: tumor description.
