# Supplementary material for: Clinicopathologic Features and Outcome of Adenocarcinoma of the Anal Canal: A Population-Based Study
Source: Int J Surg Oncol. 2020 May 13;2020:5139236. doi: 10.1155/2020/5139236 (PMC7243005; doi:10.1155/2020/5139236)
Supplement: Supplementary Materials — Supplementary Table S1: histology coding classification. Supplementary Table S2: tumor description. [file 5139236.f1.docx]

Supplemental Table 1. Histology coding classification.

| **Histology** | **ICD – O -3 Code** |
| --- | --- |
| Adenocarcinoma in situ | 8140/2 |
| Adenocarcinoma NOS | 8140/3 |
| Scirrhous adenocarcinoma | 8141/3 |
| Superficial spreading adenocarcinoma | 8143/3 |
| Carcinoma, diffuse | 8145/3 |
| Adenocarcinoma in situ in adenomatous polyp type | 8147/3, 8210/2 |
| Adenocarcinoma in adenomatous polyp | 8210/3 |
| Adenocarcinoma of anal glands, | 8215/3 |
| Adenocarcinoma with mixed subtypes, | 8255/3 |
| Papillary adenocarcinoma, NOS | 8260/3 |
| Adenocarcinoma in situ in villous adenoma | 8261/2 |
| Adenocarcinoma in villous adenoma | 8261/3 |
| Villous adenocarcinoma | 8262/3 |
| Adenocarcinoma in situ in tubulovillous adenoma | 8263/2 |
| Adenocarcinoma in tubulovillous adenoma, | 8263/3 |
| Mucinous adenocarcinoma | 8480/3 |
| Mucin-producing adenocarcinoma, | 8481/3 |
| Noninfiltrating intraductal papillary adenocarcinoma | 8503/2 |
| Intraductal papillary adenocarcinoma with invasion, | 8503/3 |
| Adenocarcinoma with squamous metaplasia | 8570/3 |
| Adenocarcinoma w cartilag. & oss. metaplas. | 8571/3 |
| Adenocarcinoma with spindle cell mataplasia | 8572/3 |
| Adenocarcinoma with apocrine metaplasia | 8573/3 |
| Adenocarcinoma with neuroendocrine differentiated 8575/3 Metaplastic carcinoma, NOS | 8574/3 |
| Hepatoid adenocarcinoma. | 8576/3 |

Supplemental Table 2: Tumor description

|  | N | % |
| --- | --- | --- |
| Anal site (Primary site, ICD-O-3 code) |  |  |
| *Anus, NOS (C21.0)* | 1001 | 47.9 |
| *Anal canal (C21.1)* | 1074 | 55.4 |
| *Cloacogenic zone (C21.2)* | 15 | 0.7 |
| Histology (ICD-0-3 code) |  |  |
| *Adenocarcinoma NOS* | 1197 | 57.3 |
| *Adenocarcinoma in tubular/villous adenoma* | 458 | 21.9 |
| *Mucin Producing Adenocarcinoma* | 368 | 17.6 |
| *All Other types of Adenocarcinoma* | 67 | 3.2 |
| Grade |  |  |
| *Grade I well differentiated; differentiated,* | 263 | 12.6 |
| *Grade II moderately differentiated; moderately differentiated; intermediate differentiation* | 858 | 41.1 |
| *Grade III poorly differentiated; differentiated* | 340 | 16.3 |
| *Grade IV undifferentiated; anaplastic* | 31 | 1.5 |
| *Cell type not determined* | 598 | 28.6 |
| SEER Stage on Presentation |  |  |
| *In-Situ* | 141 | 6.7 |
| *Localized* | 927 | 44.4 |
| *Regional* | 539 | 25.8 |
| *Distant* | 281 | 13.5 |
| *No information* | 201 | 9.6 |
